# Case Report: Anteromedial temporosphenoidal encephalocele with a clinically silent lateral bony defect in the greater wing of the sphenoid

**DOI:** 10.4103/0971-3026.57217

**Published:** 2009-11

**Authors:** Anoop Kumar Pandey

**Affiliations:** Department of Radiology, St. Paul Hospital, University of British Columbia, Canada

**Keywords:** Cerebrospinal fluid rhinorrhea, CT cisternography, encephalocele, MR imaging, temporal lobe

## Abstract

Anteromedial temporosphenoidal encephalocele is the least common type of temporal encephalocele. It commonly presents with spontaneous cerebrospinal fluid rhinorrhea in adults. This article presents the CT cisternography and MRI findings of one such case, which also had an associated clinically silent defect in the greater wing of the sphenoid on the same side.

## Introduction

Basal encephaloceles are a rare cause of spontaneous cerebrospinal fluid (CSF) rhinorrhea. They are most commonly seen in the anterior cranial fossa. Temporal encephaloceles of the middle cranial fossa are unusual but do occur and have been divided into five subtypes, with the anteromedial temporosphenoidal encephalocele being the least common type.[1] In this report, the MRI and CT scan findings of one such case are presented.

## Case Report

A 65-year-old, apparently healthy male presented with intermittent leak of clear fluid from his nostrils for the last 2 years. Initially, he had assumed that he had an attack of the common cold; however, when the leak persisted, becoming more frequent and increasing in amount, he sought medical opinion.

He gave no previous history of trauma, hypertension, headache, or meningitis. The leaking fluid was examined and was found to be CSF. Detailed clinical evaluation of the patient did not reveal any other neurological abnormality. There were no features of meningeal irritation.

**Figure 1 (A,B):**
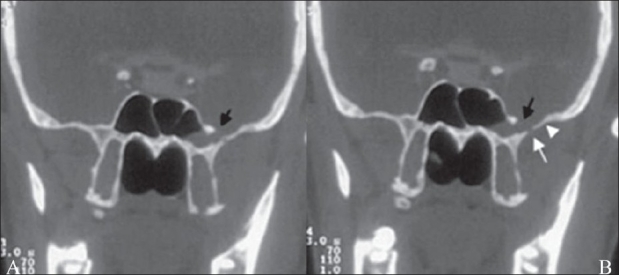
Coronal CT cisternography displays an approximately 3-mm size bony defect in the left lateral wall of the sphenoid sinus near its junction with the greater wing of the sphenoid (black arrow) along with a second, smaller, bony defect located laterally in the greater wing of the sphenoid on the left side (white arrow in B). Remodeling of the greater wing is also shown, which, as compared with the normal right side, is thinned out and bulges toward the infratemporal fossa (arrowhead in B)

CT cisternography was performed, which revealed an approximately 3-mm size defect in the left anterolateral wall of the sphenoid sinus, with a soft tissue density mass filling the adjacent part of the sphenoid sinus [Figure 1A]. Despite putting the patient in the knee–elbow position for 30 min and applying compression over the jugular veins, we could not demonstrate an active leak at the time of the examination. Another smaller bony defect was also observed laterally involving the greater wing of the left sphenoid [Figure 1B]. Remodeling of the greater wing of the left sphenoid was also seen. As compared with the normal right side, it was thinned out and bulged toward the infratemporal fossa.

**Figure 2 (A,B):**
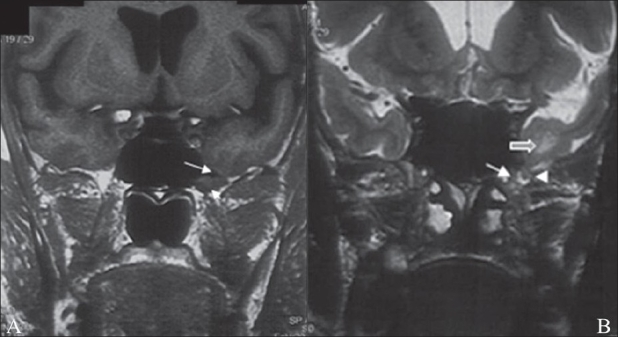
Coronal T1W MRI image (A) shows a small defect in the left inferolateral wall of the sphenoid sinus (large arrow) through which a thin tongue of anteromedial left temporal lobe (small arrow) is seen herniating into the sinus cavity. Coronal T2W image (B) shows a small cerebrospinal fluid-filled meningeal sac (arrow) herniating through the bony and dural defect (arrowhead) in the left anterolateral wall of the sphenoid sinus. This meningeal sac contains a tongue of the anteromedial temporal lobe. Focal hyperintensity is seen involving the white matter of the anteromedial temporal lobe (block arrow), which represents focal gliosis

MRI revealed a tongue of the anteromedial temporal lobe herniating through the bony defect. Abnormal T2 hyperintensity was also demonstrated in the adjacent temporal white matter. No herniation of brain or meninges was seen through the lateral defect in the greater wing of the left sphenoid [Figure 2A and B].

Taking into consideration the history of recurrent CSF rhinorrhea and the imaging findings, the patient was advised surgery. He was operated through the transnasal route. At surgery, the dural and bony defects and the associated prolapse of the anteromedial temporal lobe were confirmed. The herniated tiny lip of tissue was amputated and the dural defect was closed. The patient had complete resolution of CSF rhinorrhea and was discharged on the seventh postoperative day. He is on regular follow-up and has not shown any recurrence of symptoms over the last 2 years.

## Discussion

CSF rhinorrhea indicates that there is an abnormal communication between the intracranial subarachnoid spaces and the nasal cavity. Spontaneous CSF rhinorrhea is much less common than traumatic CSF rhinorrhea.[2] The nontraumatic, spontaneous type of CSF rhinorrhea generally has an insidious onset and can occur due to a variety of causes. Tumors involving the skull base, such as pituitary adenoma, craniopharyngioma, glioma, meningioma, olfactory neuroblastoma, and angiofibroma, are important causes of spontaneous CSF rhinorrhea.[3] Granulomatous diseases such as Wegener's granulomatosis, syphilis, and leprosy may also cause erosion of the skull base and cause CSF rhinorrhea. Suppurative sinus diseases and osteomyelitis of the skull base may lead to CSF fistulae and produce this symptom. Basal encephaloceles are uncommon causes of spontaneous CSF rhinorrhea.

These basal encephaloceles of nontraumatic etiology are more common in the anterior cranial fossa than in the middle cranial fossa. The temporal encephaloceles in the middle cranial fossa are divided into five major subtypes.[1] A lateral temporal encephalocele is a herniation of the brain matter through a defect at the pterion. It generally presents in infancy as a soft tissue mass on the lateral side of the head. An anterior temporal encephalocele results from defective development of the sphenoid wings, with consequent herniation of the anterior part of the temporal lobe into the posterior orbit. It may be seen as part of sphenoid wing dysplasia in neurofibromatosis. It presents in infancy or youth with a slowly progressive, pulsatile exophthalmos. A posteroinferior (aural) temporal encephalocele is herniation of the temporal lobe into the tympanic antrum or epitympanic recess through a defect in the tegmen tympani. It presents with CSF rhinorrhea and/or otorrhea and unilateral diminished auditory acuity. An anteroinferior temporal encephalocele results from a defect in the anteroinferior part of the floor of the middle cranial fossa through which brain tissue herniates into the infratemporal fossa. These cases typically present with complex/simple partial seizures in young adults. The least common type is the anteromedial temporal encephalocele, as shown above. In this subtype, herniation of the anteromedial part of the temporal lobe into the sphenoid sinus occurs through a defect in the lateral wall of the sphenoid sinus. These cases present in adult life with spontaneous CSF rhinorrhea. Less commonly, these patients present with headache, with or without CSF rhinorrhea. The condition is more common on the left side. It is interesting to note that all types of temporal encephaloceles have been reported to be more common in females.[1]

The development of an anteromedial temporosphenoidal encephalocele, and its presentation as CSF rhinorrhea, imply that an osteodural defect has formed in the pneumatized part of the skull base, with the development of an abnormal communication between the temporal subarachnoid space and the sphenoid sinus cavity. Some authors[2] have suggested that these osteodural defects are congenital; however, this appears unlikely, given the fact that the pneumatization of the sphenoid sinus occurs only in late childhood. Besides, all previously reported cases of such encephaloceles have been in adults, which argue against the congenital etiology. In addition, no bone/brain malformations have been reported in association with these encephaloceles,[4,5] unlike in other congenital encephaloceles. All these facts point toward an acquired etiology of these defects. Arachnoid pits have been considered to be the precursors of osteodural defects at this location. These pits are smooth, lobulated bony defects that are related to aberrant arachnoid granulations. CSF pulsation and its pressure also play an important role in the creation of these defects. This role of the CSF is evident from the pattern of the bony remodeling, with outward concave orientation of adjacent bones. This has been frequently reported previously[5] and is also seen in the present case. It is interesting to note that in many cases the rhinorrhea first occurs after activities that raise CSF pressure, such as violent blowing of the nose or stooping down to pick up heavy luggage.[3,6] Another important etiological factor is pneumatization of the inferolateral recess of the sphenoid sinus, which has been variably reported to be present in 23 and 40% of the general population in CT[5] and anatomical[7] studies, respectively. Pneumatization of the inferolateral recess is a prerequisite if an osteodural defect at this location is to present as CSF rhinorrhea.[8]

The present case is unique as two bony defects were definitely documented. One defect was adjacent to the foramen rotundum at the junction of the body and the greater wing of the sphenoid bone, and the encephalocele had developed through this; the other, smaller, defect was located laterally in the greater wing of the sphenoid. Another interesting feature in this case was that, as compared with the contralateral side, there was significant remodeling of the greater wing of the left sphenoid in the form of thinning and bowing toward the infratemporal fossa, a finding that supports the theory that long-standing outward CSF pressure plays an important role in the development of these encephaloceles.

All cases of anteromedial temporal encephaloceles reported in the literature have been treated surgically. Surgical treatment is necessary as the CSF rhinorrhea seldom resolves on its own; also, when there is continuous or recurrent CSF rhinorrhea, there is a high risk of meningitis. Both transnasal[9–13] and transcranial[4,6,14–18] surgical approaches have been used with equal success. In general, and as in the present case, the transnasal route is preferred for smaller defects; larger defects are treated via the transcranial route.[14] Imaging plays an important role in the preoperative identification of the site and etiology of the CSF leak. CT cisternography may depict the site of the bony defect and the CSF leak. Owing to the excellent soft tissue contrast that it provides, MRI is invaluable for preoperative delineation of the contents of the herniation, which has an important bearing on treatment planning, particularly if endoscopic surgery is being planned.[8]

In conclusion, anteromedial temporosphenoidal encephalocele is a rare cause of spontaneous CSF rhinorrhea in adults and imaging with CT/MRI is particularly useful for its confident preoperative diagnosis.
